# Simulation-based education in undergraduate psychiatry: a focused review and interpretive analysis of clinical competence representation

**DOI:** 10.1186/s12909-026-09885-2

**Published:** 2026-07-06

**Authors:** Francisco Rodríguez Pulido, Ángeles Rodríguez García, Nayra Caballero Estebaranz

**Affiliations:** 1 Psychiatry area, School Medicine, University of La Laguna, Tenerife, Canary Island Spain; 2 Department of Nursing, University of La Laguna, Tenerife, Canary Island Spain; 3 Department of Clinical Neuropsychology, Canary Islands Health Research Institute Foundation, Santa Cruz de Tenerife, Spain

**Keywords:** Simulation-based education, Psychiatry education, Undergraduate medical education, Standardized patients, Clinical competence, Competency-based medical education, Clinical reasoning, Psychiatric assessment, Medical simulation, Competence representation

## Abstract

**Background:**

Simulation-based education has become an established component of medical training and is increasingly used in undergraduate psychiatry. While previous studies have primarily examined its effectiveness for developing clinical skills, less attention has been given to how different dimensions of psychiatric competence are represented within simulation-based educational and assessment frameworks. This review explores the representation of behavioural, relational and integrative competencies in simulation-based undergraduate psychiatric education.

**Methods:**

A focused review was conducted following PRISMA principles. Electronic databases were searched to identify studies examining simulation-based education in undergraduate psychiatry. Eligible studies involved undergraduate medical students and the use of standardized or simulated patients in psychiatric teaching or assessment. Data were analysed using a narrative and interpretive synthesis informed by contemporary literature on professional competence, clinical reasoning and psychiatric practice.

**Results:**

Four studies met the inclusion criteria. Across the reviewed literature, behavioural competencies, including interviewing skills, information gathering and psychopathological assessment, were consistently represented. Relational competencies, such as empathy, communication and therapeutic engagement, were also explicitly addressed. In contrast, integrative competencies involving formulation, contextual understanding, biopsychosocial synthesis and clinical judgement were rarely operationalised as explicit assessment constructs. Although integrative reasoning appeared to be implicitly required within complex clinical encounters, it remained comparatively less visible within published educational and assessment frameworks.

**Conclusions:**

Simulation-based education contributes to the development of important clinical skills in undergraduate psychiatry. The findings suggest that behavioural and relational competencies are consistently represented within simulation-based educational and assessment approaches, whereas integrative competence is less frequently operationalised as an explicit construct. The concept of competence representation offers a useful perspective for understanding how psychiatric competence is conceptualised and assessed and may inform future developments in simulation-based psychiatric education.

## Introduction

Simulation-based education has become an increasingly important component of undergraduate medical education and is now widely used across multiple clinical disciplines to support the development of communication skills, clinical reasoning and professional competence [1, 2]. Within psychiatry, simulation most commonly involves the use of standardized patients who recreate authentic clinical scenarios in controlled educational environments, allowing learners to practise interviewing, assessment and decision-making skills while minimising risks to patients.

The educational value of simulation is particularly relevant in psychiatry because the clinical interview occupies a central position within psychiatric practice. Unlike many areas of medicine in which diagnosis may rely heavily on laboratory investigations or imaging findings, psychiatric assessment depends primarily upon communication, observation and the interpretation of subjective experiences. Through the interview, clinicians gather information, establish therapeutic relationships, formulate diagnostic hypotheses and develop treatment plans. Consequently, simulation provides opportunities not only to teach clinical skills but also to recreate many of the complex interpersonal processes that characterise psychiatric practice.

A substantial body of literature has demonstrated the effectiveness of simulation-based education in improving communication skills, learner confidence, interview structure and preparedness for clinical encounters [1, 2]. Standardized patients and Objective Structured Clinical Examinations (OSCEs) have become established components of undergraduate psychiatric training and are frequently used to assess learner performance in a structured and reproducible manner. Previous reviews have generally focused on educational effectiveness, learner satisfaction and skill acquisition, consistently reporting positive educational outcomes associated with simulation-based learning.

However, comparatively less attention has been devoted to a more fundamental question concerning the nature of psychiatric competence itself and how it is represented within educational assessment frameworks. This issue is particularly relevant within competency-based medical education (CBME), where assessment systems play a central role in defining, evaluating and communicating professional competence [3, 4]. Although considerable attention has been given to the development of competency frameworks, less discussion has focused on how different dimensions of competence become visible within educational assessment practices.

Psychiatric competence presents distinctive challenges in this regard. Effective psychiatric practice requires more than the performance of observable clinical behaviours. In addition to communication and interviewing skills, clinicians must integrate symptoms, subjective experiences, interpersonal dynamics and contextual influences into coherent understandings that guide clinical decision-making. Psychiatric understanding frequently develops through the synthesis of biological, psychological and social information and often occurs under conditions of uncertainty [5, 6]. Consequently, competence involves not only what clinicians do, but also how they organise, interpret and apply clinical information.

Drawing upon contemporary perspectives on professional competence, psychiatric competence may be conceptualised as comprising at least three interrelated domains. Behavioural competence refers to observable clinical actions, including information gathering, symptom exploration and interview organisation. Relational competence encompasses empathy, therapeutic engagement and the capacity to establish collaborative clinical relationships. Integrative competence involves the formulation of clinically meaningful understandings through the synthesis of biological, psychological and social information [7]. These perspectives are consistent with biopsychosocial approaches to health and with broader models emphasising the contribution of lifestyle, environment and social context to health outcomes [5, 7].Although these domains are closely interconnected in practice, they may differ in the extent to which they are explicitly represented within assessment systems.

This distinction raises important questions concerning construct representation. Assessment methods necessarily operationalise particular aspects of competence in order to make them observable and measurable. However, competencies that are more readily observable may achieve greater visibility within assessment frameworks than competencies that depend upon synthesis, contextual interpretation or clinical judgement [8]. Consequently, assessment systems may represent different dimensions of psychiatric competence with varying degrees of explicitness.

For the purposes of this review, competence representation refers to the extent to which particular domains of psychiatric competence are explicitly specified, operationalised and assessed within educational and assessment frameworks. Importantly, the issue is not whether integrative reasoning occurs during simulation-based learning. Complex psychiatric scenarios are likely to require such reasoning. Rather, the question is whether these processes are explicitly represented, measured and evaluated within published assessment approaches.

The present study therefore aims to examine how clinical competence is represented within simulation-based education in undergraduate psychiatry. Specifically, it explores the extent to which behavioural, relational and integrative domains of competence are reflected within assessment frameworks reported in the published literature. By combining a focused systematic review with an interpretive synthesis, the review seeks not only to summarise available evidence but also to address an important and relatively underexplored conceptual question concerning the relationship between psychiatric competence, simulation-based learning and educational assessment.

Recent educational literature continues to emphasise the central role of psychiatric interviewing and mental status examination in undergraduate psychiatric training. A recent scoping review highlighted the diversity of instructional approaches used to teach these skills and underscored their importance as foundational components of psychiatric competence [9]. Despite this growing educational interest, less attention has been paid to how broader dimensions of competence are represented within simulation-based learning and assessment frameworks.

The aim of this focused review was not only to examine the use of simulation-based education in undergraduate psychiatry, but also to explore how different dimensions of clinical competence are represented within simulation-based educational and assessment frameworks. Particular attention was given to the visibility of behavioural, relational and integrative competencies, and to the implications of their representation for psychiatric education.

## Methods

### Study design

This study was conducted as a focused systematic review with narrative and interpretive synthesis following the Preferred Reporting Items for Systematic Reviews and Meta-Analyses (PRISMA 2020) guidelines [10].

A focused systematic review was considered appropriate because the objective extended beyond evaluating the educational effectiveness of simulation-based psychiatry training and sought to examine how psychiatric clinical competence is represented within published assessment frameworks. This approach allowed the integration of empirical findings with a theoretically informed analysis of competence representation.

### Search strategy

Electronic searches were conducted in PubMed/MEDLINE, Scopus and Web of Science. Search terms combined concepts related to psychiatry, simulation-based education and undergraduate medical training. A representative search strategy included the following search string: (“psychiatry” OR “psychiatric education”) AND (“simulation” OR “simulation-based education” OR “standardized patient” OR “simulated patient”) AND (“medical students” OR “undergraduate medical education”) AND (“competence” OR “assessment”Search terms were adapted as appropriate for each database.

### Study selection and data extraction

Titles and abstracts were screened for relevance according to predefined eligibility criteria. Full-text articles were subsequently assessed to determine final eligibility. Studies were required to involve undergraduate medical students and the use of simulation-based educational approaches in psychiatry, including standardized or simulated patients. Articles focusing exclusively on postgraduate training, non-psychiatric simulation, or lacking educational or assessment outcomes were excluded.

Data extraction focused on study characteristics, educational context, participant characteristics, simulation format, assessment approaches and reported educational outcomes. Particular attention was given to the extent to which behavioural, relational and integrative dimensions of competence were explicitly represented within assessment frameworks.

Study screening and eligibility assessment were undertaken by the authors. Any uncertainties regarding study eligibility or interpretation were resolved through discussion and consensus among the research team. Data extraction was conducted using a structured framework focusing on study characteristics, simulation format, assessment approaches and reported competence domains.

### Eligibility criteria

Studies were eligible for inclusion if they:

- involved undergraduate medical students;
- examined simulation-based education within psychiatry;
- included standardized patients, simulated patients or equivalent simulation approaches;
- reported educational, assessment or competency-related outcomes.

Studies were excluded if they:

- focused exclusively on postgraduate or specialist psychiatric training;
- did not involve psychiatry-specific educational activities;
- lacked relevant educational or assessment outcomes;
- were editorials, commentaries or conference abstracts.

### Interpretive synthesis

Given the heterogeneity of study designs, interventions and outcome measures, formal meta-analysis was considered inappropriate. Narrative and interpretive synthesis was therefore undertaken following established guidance for narrative synthesis in health research.

The synthesis was primarily framework-informed and deductive in orientation.

Drawing upon literature relating to professional competence, clinical reasoning and competency-based medical education [3, 4, 7], findings were analysed according to three competence domains:

- Behavioural competence.
- Relational competence.
- Integrative competence.

Behavioural competence refers to observable clinical actions such as information gathering, symptom exploration and interview organisation. Relational competence encompasses empathy, rapport and therapeutic engagement. Integrative competence refers to the formulation of clinically meaningful understandings through the synthesis of biological, psychological and social information [5, 8].

Studies were examined to determine which domains were explicitly represented within assessment frameworks, which were only implicitly addressed and which were not reported.

Themes and interpretations were developed through iterative comparison across studies and discussion among the authors. The objective was not to infer actual learner cognition but rather to examine how competence was represented within published assessment approaches.

### Reporting

The review was conducted and reported in accordance with PRISMA 2020 recommendations [10]. A completed PRISMA checklist is provided as supplementary material.

## Results

### Study selection

The database search identified 143 records. After removal of duplicates, 118 records underwent title and abstract screening. Twenty-one articles were retrieved for full-text review. Following application of the predefined eligibility criteria, four studies were included in the final synthesis.

Following reassessment of the full-text articles against the predefined eligibility criteria, one study was excluded from the final synthesis because, although relevant to simulation-based education, it did not specifically investigate undergraduate psychiatric simulation as defined by the inclusion criteria. The final synthesis therefore comprised four studies.

**Fig. 1:**
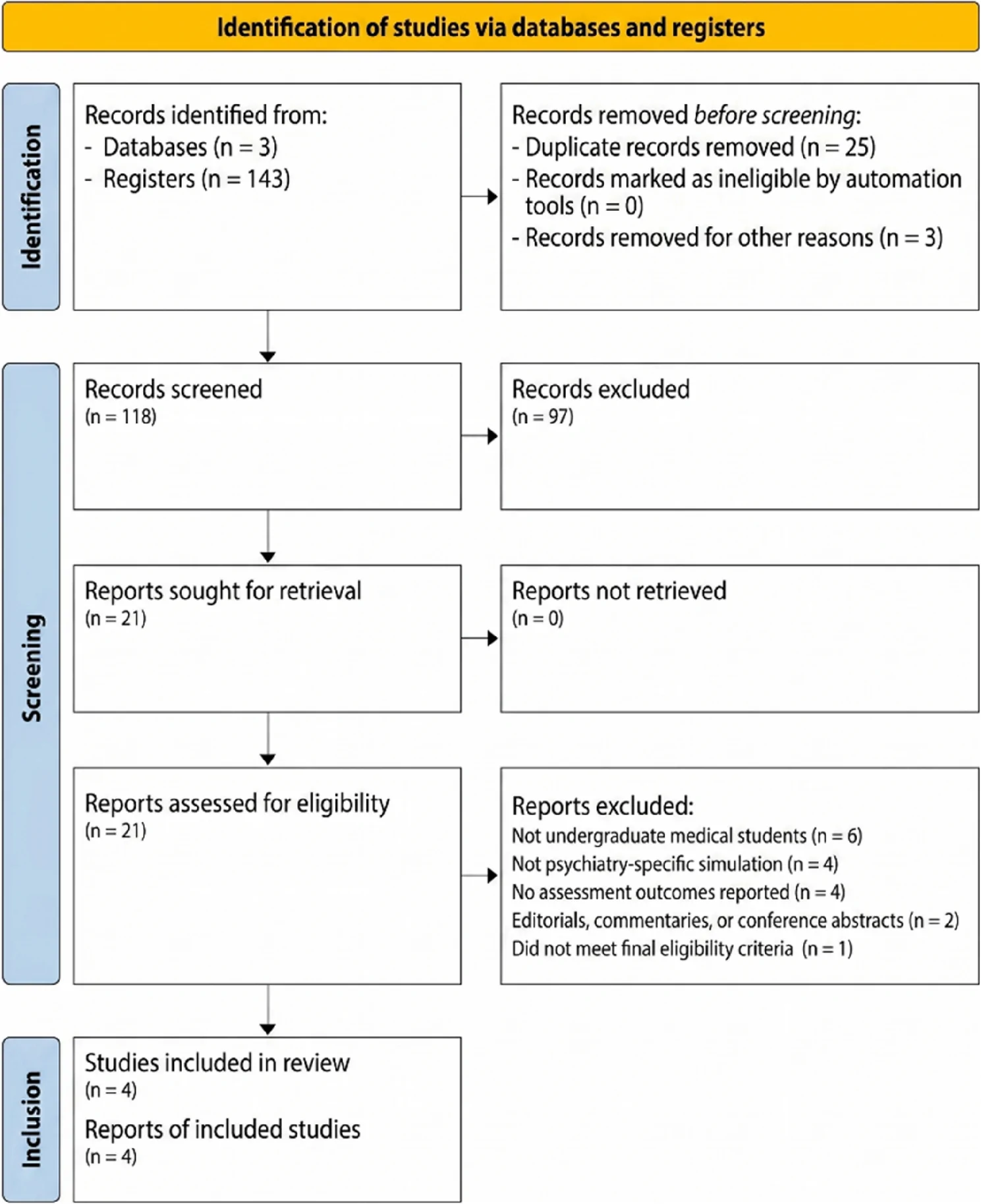
PRISMA 2020 flow diagram of study selection of studies examining simulation-based education in undergraduate psychiatry

The study selection process is presented in Fig. 1.

The four included studies encompassed a range of educational designs, participant groups and assessment approaches[11–14]. Sample sizes ranged from 41 to 469 participants, reflecting substantial variation in study scope and design.

Most interventions employed standardized or simulated patients to recreate psychiatric interviews and communication challenges in controlled educational environments.

The studies were conducted primarily in North America and Europe and included quasi-experimental, descriptive and educational evaluation designs. Assessment approaches varied considerably and included structured checklists, global rating scales, standardized patient assessments, survey measures and qualitative analyses.

Study characteristics are summarised in Table 1.

**Table 1:** Characteristics of included studies

	Country	Study Design	Participants	Simulation Format	Main Outcomes
Bennett et al. (2006) [11]	USA	Quasi-experimental educational study	Third-year medical students during psychiatry clerkship	Standardized patient examination (Psychiatry Clinical Standardized Patient Examination)	Improved interviewing skills, professionalism, interpersonal communication and positive clinical ratings
Wear & Varley (2008) [12]	USA	Critical review	Not applicable	Standardized patients in psychiatric simulation	Analysis of strengths and limitations of simulation for evaluating empathic communication
Brenner (2009) [13]	USA	Narrative review	Not applicable	Standardized patients in psychiatric education	Examination of uses and limitations of standardized patients for psychiatric teaching and assessment
Siemerkus et al. (2023) [14]	Switzerland	Educational evaluation with survey methodology	41 third-year medical students, 6 actors, 8 lecturers	Standardized patient interviews with supervised feedback	Improved psychiatric interviewing, psychopathological assessment and empathic communication; high perceived authenticity and acceptability

### Representation of competence domains

Across the included studies, three competence domains were identified through the interpretive synthesis:

- behavioural competence;
- relational competence;
- integrative competence.

Although all three domains were potentially relevant to psychiatric practice, their representation within assessment frameworks varied considerably.

Behavioural competencies were most frequently operationalised and directly assessed. Relational competencies were represented less consistently and were often evaluated through global judgements or qualitative observations. Integrative competencies were rarely operationalised as explicit assessment constructs.

The distribution of competence domains across studies is summarised in Table 2.

**Table 2:** Representation of competence domains across included studies

Study	Behavioural Competence	Relational Competence	Integrative Competence
Bennett et al. (2006) [11]	Explicitly represented through interviewing performance, information gathering and clinical communication skills	Explicitly represented through professionalism and interpersonal communication	Not explicitly assessed or operationalised
Wear & Varley (2008) [12]	Explicitly represented through observable communication behaviours	Explicitly represented through empathic communication	Implicitly discussed through concerns regarding authentic understanding and empathy
Brenner (2009) [13]	Explicitly represented through assessment of discrete psychiatric skills	Explicitly represented through empathic engagement and interpersonal processes	Implicitly addressed through discussion of limitations in assessing complex interpersonal and psychotherapeutic competencies
Siemerkus et al. (2023) [14]	Explicitly represented through psychiatric history taking and psychopathological assessment	Explicitly represented through non-judgmental and empathic communication	Implicitly required during case discussion but not formally assessed as a separate construct

### Behavioural competence

Behavioural competence was the most consistently represented domain across studies. Assessment approaches focused primarily on observable interviewing behaviours, including symptom exploration, information gathering, communication techniques and interview organisation.

Structured checklists and OSCE-based rating systems were the most commonly reported assessment methods. Improvements in communication performance, interviewing skills and learner confidence were frequently reported outcomes.

### Relational competence

Relational competence was represented through constructs such as empathy, rapport, therapeutic engagement and active listening. Several studies emphasised the importance of interpersonal processes within psychiatric encounters and reported positive effects of simulation on students’ confidence in managing emotionally complex interactions.

However, relational competence was less consistently operationalised than behavioural competence and was frequently assessed through global judgements rather than clearly defined performance indicators.

### Integrative competence

Integrative competence was the least explicitly represented domain.

None of the included studies operationalised biopsychosocial formulation or integrative clinical reasoning as clearly defined and independently assessed competence domains. Nevertheless, several simulation scenarios appeared sufficiently complex to require learners to synthesise symptoms, contextual influences and interpersonal information in order to understand the clinical situation.

Importantly, these findings should not be interpreted as evidence that integrative reasoning was absent from simulation-based learning activities. Rather, they suggest that such reasoning was infrequently specified, measured or reported as an explicit assessment construct within the published literature.

### Construct visibility

A recurring pattern across studies was the predominance of assessment approaches focused on observable performance.

Behavioural competencies demonstrated high visibility within assessment frameworks. Relational competencies showed moderate visibility, whereas integrative competencies were largely implicit and rarely operationalised independently.

This pattern suggests differences in construct visibility rather than differences in educational importance. Integrative reasoning may occur during simulation encounters but remain difficult to identify when assessment systems prioritise directly observable behaviours.

## Discussion

### Principal findings

The present review examined how clinical competence is represented within simulation-based education in undergraduate psychiatry. Beyond questions of educational effectiveness, the findings suggest that different domains of competence achieve different levels of visibility within published assessment frameworks. Behavioural competencies were most frequently operationalised and assessed, relational competencies demonstrated intermediate visibility, whereas integrative competencies were less commonly represented as explicit assessment constructs.

These findings should not be interpreted as evidence that integrative reasoning is absent from simulation-based learning. Rather, they suggest that formulation, contextual understanding and integrative clinical reasoning are less frequently specified, operationalised and evaluated within the assessment approaches reported in the reviewed literature. The distinction therefore concerns competence representation rather than competence acquisition.

Importantly, these interpretations emerge from a narrative and interpretive synthesis of heterogeneous educational evidence, an approach that seeks to identify conceptual patterns across studies rather than aggregate quantitative outcomes [15]. This distinction is consistent with contemporary conceptions of professional competence, which emphasise that competence extends beyond the execution of observable tasks and includes the integration of knowledge, judgement, contextual understanding and reflective decision-making [16].

### Competence representation and visibility

Assessment systems do not merely measure competence; they also contribute to making particular forms of competence visible within educational practice. Competencies that are explicitly assessed tend to receive greater attention within curricula, teaching activities and learner preparation. Consequently, competence representation may be understood not only as a psychometric issue but also as an educational one.

From a construct validity perspective, assessment systems inevitably privilege those aspects of competence that can be most readily specified, observed and measured. Consequently, visibility within assessment frameworks may not necessarily correspond to educational importance [8, 17].

The reviewed studies suggest that behavioural competencies may be more readily represented because they are directly observable and can be operationalised through structured assessment criteria. Integrative competencies, by contrast, often involve processes of synthesis, contextual interpretation and clinical judgement that may be less immediately visible within assessment encounters.

The challenge may therefore not be that integrative competence is absent from psychiatric education, but that it remains comparatively less visible within the frameworks through which competence is recognised and evaluated. This distinction may help explain why some dimensions of psychiatric competence appear underrepresented within published assessment systems despite their recognised importance in clinical practice.

### Psychiatry as an integrative clinical practice

The significance of this issue becomes particularly apparent when viewed from the perspective of psychiatric practice. Psychiatric competence extends beyond the identification of symptoms or the performance of interviewing behaviours. Clinical understanding frequently depends upon the integration of subjective experiences, personal history, interpersonal relationships and social context into coherent formulations capable of guiding therapeutic action.

Psychiatric assessment frequently requires clinicians to move beyond symptom description towards the interpretation of subjective experiences and their relationship to personal, interpersonal and social contexts. Formulation occupies a central position within this process because it allows clinicians to organise observations, experiences and contextual information into clinically meaningful explanations that support diagnosis, treatment planning, risk assessment and prognostic judgement.

This interpretive dimension has long occupied a central place within psychiatric theory. Classical psychopathology emphasised the importance of understanding subjective experience within meaningful contexts rather than relying exclusively on behavioural description [18, 19].

In this sense, formulation functions as a bridge between observation and therapeutic decision-making. Psychiatric practice often requires clinicians to work under conditions of uncertainty and to integrate information across biological, psychological, relational and social domains. Contemporary recognition of biopsychosocial influences and social determinants of health further reinforces the importance of contextual and integrative forms of understanding [5, 6].

Consequently, psychiatry provides a particularly informative context in which to examine questions concerning competence representation.

### Educational implications

The findings of this review raise broader questions concerning the relationship between assessment and professional formation. Assessment frameworks inevitably reflect assumptions regarding what constitutes competent clinical practice (Table 3). Competencies that are consistently represented within assessment systems become more visible within educational programmes and may influence how learners understand professional expectations.

**Table 3:** Conceptual framework for the representation of behavioural, relational and integrative competence in simulation-based psychiatry

Competence Domain	Primary Educational Focus	Typical Assessment Approaches	Visibility Within Current Literature
Behavioural	Interviewing, symptom exploration, information gathering	Checklists, OSCE stations, performance ratings	High
Relational	Empathy, rapport, therapeutic engagement	Global ratings, standardized patient feedback, communication scales	Moderate to High
Integrative	Formulation, contextual understanding, biopsychosocial synthesis, clinical judgement	Rarely operationalised; occasionally inferred through discussion and reflection	Low

Assessment frameworks may therefore shape not only what is evaluated, but also what learners perceive as central to competent psychiatric practice. From this perspective, competence representation becomes closely linked to how psychiatric expertise is conceptualised and communicated.

Although not eligible for inclusion in the final synthesis because it involved postgraduate psychiatric trainees rather than undergraduate medical students, Klamen and Yudkowsky described the use of standardized patients to provide formative feedback in an introductory psychotherapy course. Their work highlights the continuing educational importance of relational, reflective and psychotherapeutic competencies, suggesting that dimensions of competence extending beyond observable interviewing behaviours remain relevant across different stages of psychiatric training [20]. Future undergraduate simulation programmes may benefit from exploring how such competencies can be represented more explicitly within assessment frameworks.

The challenge for psychiatric education is not to choose between behavioural performance and integrative reasoning, but rather to consider how assessment systems can represent the relationship between observation, formulation, clinical judgement and therapeutic action in ways that remain educationally feasible and psychometrically robust.

Recent evidence also supports the educational value of simulation-based psychiatric training for developing communication and interviewing skills among medical students. Amsalem and colleagues demonstrated that even brief simulation-based interventions can improve psychiatric and communication competencies, reinforcing the continuing importance of experiential learning approaches within undergraduate psychiatry curricula [14, 21]. These findings are consistent with the present review and further support the role of simulation as a valuable component of psychiatric education.

Future simulation-based assessments may benefit from combining behavioural observation with explicit evaluation of formulation and reasoning processes. Following simulated encounters, learners could be asked to construct brief biopsychosocial formulations, justify diagnostic hypotheses, identify contextual contributors to distress and explain the reasoning underlying their clinical decisions.

### Strengths, limitations and future directions

A strength of this review is its combination of systematic review methodology with an interpretive analysis focused on competence representation, a perspective that has received relatively limited attention within psychiatric education research. The study also proposes a distinction between competence acquisition and competence representation, which may provide a useful framework for future investigations.

Several limitations should be considered when interpreting the findings. First, the evidence base was relatively small, with only four studies meeting the final inclusion criteria. Although these studies were directly relevant to the review question, the limited number of publications restricts the breadth of conclusions that can be drawn regarding simulation-based psychiatric education.

Second, substantial heterogeneity was observed across studies in terms of educational context, simulation design, assessment methods and outcome measures. This heterogeneity precluded quantitative synthesis and supported the decision to undertake a narrative and interpretive approach.

Third, publication bias cannot be excluded. Studies reporting positive educational outcomes may be more likely to reach publication than studies reporting neutral or negative findings, potentially influencing the available evidence base.

Fourth, the review relied on published descriptions of educational interventions and assessment approaches. Consequently, interpretations regarding competence representation are constrained by the level of detail reported by study authors. It is possible that integrative competence was addressed within simulation activities but not explicitly described in publications or assessment instruments.

Fifth, the interpretive synthesis inevitably involved an element of analytical judgement. Although the analysis was guided by a predefined conceptual framework and informed by established literature on professional competence, clinical reasoning and psychiatric practice, alternative interpretations remain possible.

Finally, an important distinction should be emphasised. This review examined competence representation within published educational and assessment frameworks rather than learners’ actual reasoning processes. Conclusions therefore relate to how competence is operationalised and evaluated and should not be interpreted as evidence regarding the presence or absence of integrative reasoning during simulation-based learning activities.

Future research should explore approaches capable of representing behavioural, relational and integrative dimensions of competence in complementary ways. Particular attention should be given to how formulation, contextual understanding and clinical judgement can be incorporated into simulation-based assessment while maintaining validity, reliability and educational feasibility.

Despite these limitations, the review provides a transparent and theoretically informed examination of how psychiatric competence is represented within simulation-based education and identifies directions for future educational development and research.

## Conclusions

Simulation-based education appears to be an effective strategy for developing key clinical skills in undergraduate psychiatry, particularly in relation to communication, interviewing and therapeutic engagement. The available evidence suggests that simulation contributes positively to learner preparedness and supports competencies that are central to psychiatric practice.

At the same time, the review indicates that different domains of psychiatric competence achieve different levels of visibility within published assessment frameworks. Behavioural competencies are most frequently represented and assessed, whereas integrative competencies, including formulation, contextual understanding and biopsychosocial synthesis, are less commonly operationalised as explicit assessment constructs.

Importantly, these findings should not be interpreted as evidence that integrative competence is absent from psychiatric education. Rather, they suggest that such competencies may be less visible within the frameworks through which competence is recognised and evaluated.

The concept of competence representation offers a useful perspective for examining the relationship between psychiatric competence and educational assessment. Greater attention to formulation, contextual understanding and clinical judgement may contribute to assessment approaches that more fully reflect the integrative nature of psychiatric expertise.

Ultimately, one of the principal challenges for psychiatric education is not only to assess what learners do during clinical encounters, but also to make visible how they transform observations, relationships and contextual information into clinically meaningful understanding that guides therapeutic action.

## Data Availability

All data analysed during this study are included in this published article.
